# Work productivity is associated with disease activity and functional ability in Italian patients with early axial spondyloarthritis: an observational study from the SPACE cohort

**DOI:** 10.1186/s13075-016-1162-3

**Published:** 2016-11-16

**Authors:** Manouk de Hooge, Roberta Ramonda, Mariagrazia Lorenzin, Paola Frallonardo, Leonardo Punzi, Augusta Ortolan, Andrea Doria

**Affiliations:** Rheumatology Unit, Department of Medicine –DIMED, University of Padova, Via Giustiniani 2, 35128 Padova, Italy

**Keywords:** Spondyloarthritis, Work, Back pain, Disease activity

## Abstract

**Background:**

Spondyloarthritis often affects young people, typically in their working years. The aim of our study was to investigate work productivity and its relationship with disease activity and physical functioning in Italian patients with axial spondyloarthritis (axSpA) with chronic back pain (CBP) for ≥3 months and ≤2 years, and onset  < 45 years of age.

**Methods:**

Baseline absenteeism, presenteeism, work productivity loss (assessed by the Work Productivity and Activity Impairment questionnaire (WPAI)), and disease activity (assessed by the Bath Ankylosing Spondylitis Disease Activity Index (BASDAI)/Ankylosing Spondylitis Disease Activity Score (ASDAS)) and functional ability (assessed by the Bath Ankylosing Spondylitis Disease Functional Index (BASFI)) of patients with axSpA (rheumatologist’s diagnosis) included in the Italian section of the Spondyloarthritis Caught Early (SPACE) cohort were collected. Multivariate linear regression analysis was used to evaluate the associations between work productivity and disease activity/physical functioning.

**Results:**

Absenteeism in 51 patients with axSpA was low (8.3 %). A decrease in work productivity was related to an increase in disease activity. Disease activity was strongly correlated with absenteeism (*p* < 0.01), presenteeism (*p* < 0.01) and work productivity loss (*p* < 0.001). In addition, decreased work productivity was related to a decrease in functional ability. Physical functioning was correlated with absenteeism (*p* < 0.001), presenteeism (*p* < 0.05) and work productivity loss (*p* < 0.001).

**Conclusions:**

Impairment of work productivity was correlated with disease activity and physical functioning in Italian patients with axSpA with CBP for ≥3 months and ≤2 years, with onset <45 years of age.

## Background

Spondyloarthritis (SpA) are a group of chronic inflammatory rheumatic diseases that share several clinical characteristics. They can be subdivided into axial SpA (axSpA) or peripheral SpA depending on the predominant musculoskeletal site involved [1]. Patients with axSpA can be further subdivided into two groups: patients with radiographic evidence of sacroiliitis (ankylosing spondylitis, AS) or without radiographic sacroiliitis (non-radiographic axSpA, nr-axSpA). Patients with axSpA often suffer from impaired function, activity limitations and decreased health-related quality of life (HRQoL) [2–4]. AxSpA usually occurs during young adulthood, before the third decade [5]; thus, function impairment and activity limitations can have important socioeconomic consequences because they affect patients who have just started working. The work participation of axSpA patients with longstanding disease has previously been reported in several studies, which showed correlation between decreased work productivity and increased disease-related sick leave [6–8]. Limitations in physical functioning are also strongly associated with work restrictions in patients with axSpA [2, 6]. Besides limitations in work productivity when performing paid work, patients with axSpA also seem to be limited in performing daily activities such as taking care of the family, studying or housekeeping [6].

A study focusing on three European countries (Netherlands, Belgium and France) showed that the work status and productivity of patients with AS differ among these countries [9], decreasing the generalizability of the correlation between work productivity and disease activity or physical functioning in patients with axSpA as identified in previous studies. The prevalence of axSpA in Italy is 1.06 % [10] and the prevalence of nr-axSpA is estimated to be 0.35 % [11]. Few data on work productivity in Italian patients with axSpA are available [12]; it is therefore important to get a clear picture on how this disease impacts the work productivity. The aim of our study was to investigate work productivity and its relationship with disease activity and physical functioning in Italian patients with short-term chronic back pain (CBP) who participated in the Italian section of the Spondyloarthritis Caught Early (SPACE) cohort. In particular presenteism, absenteeism, and work productivity loss (WPL) were considered. Presenteism represents the reduced work productivity due to disease; absenteeism is the percentage of hours missed due to disease; WPL gives an indication of the total work impairment due to disease.

## Methods

### Patients

Patients who were at least 16 years old, suffering from CBP (≥3 months, ≤2 years, onset  < 45 years of age) of unknown origin and referred to a rheumatologist were included in the Italian section of the SPACE cohort. This is an ongoing observational cohort study, which was originally launched at the Leiden University Medical Centre (LUMC) in January 2009.

In March 2012 the SpA study group of the Rheumatology Unit at the University of Padua opened an Italian branch of the SPACE cohort. Eligible patients underwent physical examination, laboratory tests, radiographic imaging and magnetic resonance imaging (MRI) of the sacroiliac joints and spine according to a standardized protocol. In addition, patients completed questionnaires on disease activity, physical functioning, pain and impairment due to the disease, and work productivity. The SPACE cohort has been extensively described in a previous publication [13]. An experienced rheumatologist diagnosed axSpA. Only baseline data were used in these analyses. At this time point, all patients were treated with non-steroidal anti-inflammatory drugs. No patients were treated with synthetic or biological disease modifying anti-rheumatic drugs.

### Questionnaires

The Work Productivity and Activity Impairment (WPAI) questionnaire is validated to assess the impact of a disease (here axSpA) on work productivity and on other daily activities [14, 15]. The Italian version of the WPAI was used to obtain the data in our study (from http://www.reillyassociates.net/WPAI_Translations.html) [16]. By using six items, the questionnaire measures: (Question (Q)1) patients’ employment status; (Q2) number of hours missed at work due to axSpA; (Q3) number of hours missed at work due to due to other reasons; (Q4) number of hours worked effectively according to the patient’s own judgment; (Q5) degree of disease influence on work productivity, according to the patient; and (Q6) degree of disease influence on activities not related to work, according to the patient [13].

The WPAI questionnaire allows the calculation of absenteeism, presenteeism and work productivity loss (WPL). Presenteeism represents the reduced work productivity due to disease, which is rated on a 0–100 % numeric scale, and is calculated as the degree of disease influence on work productivity/10 (question (Q)5). Absenteeism is the percentage of hours missed due to disease (and not due to other reasons) and is calculated as follows the number of hours missed at work due to axSpA (Q2)/(number of hours missed at work due to axSpA (Q2) + number of hours worked effectively (Q4)) [14]. The WPL gives an indication on the total work impairment due to disease and is derived from presenteeism and absenteeism as follows: absenteeism + ((1-Absenteism) × (Presenteeism)). All scores are to be multiplied by 100 to be expressed as percentages. The higher the presenteeism, absenteeism or WPL scores, the greater the work impairment is due to disease.

It has to be underlined that as patients could do both paid work and other daily activities, they reported separately on the WPAI items for paid work (in the form of employee or self-employment) and/or daily activities (in the form of domestic work, studying, voluntary work or taking care of family members). The disease activity and physical functioning of patients were assessed with self-reported questionnaires: the Bath Ankylosing Spondylitis Disease Activity Index (BASDAI), the Bath Ankylosing Spondylitis Functional Index (BASFI) and the Ankylosing Spondylitis disease activity score (ASDAS), which combines items from the BASDAI, patient global assessment and serum C-reactive protein (CRP) [17]. Four ASDAS categories were defined: inactive disease (ASDAS <1.3), moderate disease activity (ASDAS ≥1.3 and <2.1), high disease activity (ASDAS ≥2.1 and ≤3.5) and very high disease activity (ASDAS >3.5) [18]. BASDAI and BASFI continuous scores were converted into dichotomous scores. BASDAI <4 indicated low disease activity and BASDAI ≥4 indicated high disease activity [17]. As there is no BASFI score for impaired and unimpaired physical functioning, we arbitrarily choose a BASFI score ≥2.5 to indicate impaired physical functioning.

### Statistical analyses

Patient characteristics were reported by descriptive statistics. The Mann-Whitney test was used to evaluate the association between work productivity and disease activity (evaluated by BASDAI) and physical functioning. The Kruskal-Wallis test was used to evaluate the relationship between work productivity and the four ASDAS states. Multivariate linear regression analyses were performed to evaluate the association of BASDAI, ASDAS and BASFI continuous scores with presenteeism, absenteeism and WPL, adjusted for age, gender, HLA-B27 positivity and duration of CBP. SPSS 21 was used to perform statistical analysis.

## Results

Out of 51 patients enrolled in the study, 100 % fulfilled the ASAS criteria for axSpA; 4 (7.8 %) were diagnosed as having AS and 47 (92.2 %) as having nr-axSpA. Mean age at CBP onset was 29.7 (SD ± 8.7) years, 21 patients (41.2 %) were men and 16 patients (31.4 %) were HLA-B27 positive. All 51 patients had at least two features of SpA and the maximum disease activity and physical functioning scores were 9.1 (BASDAI), 4.2 (ASDAS) and 8.9 (BASFI). Other patient characteristics, including work status and mean scores for disease activity and physical functioning, are reported in Table 1. A detailed description of patient characteristics has been published before [19].

**Table 1:** Baseline characteristics of Italian patients with axSpA included in the SPACE cohort (n = 51)

Characteristic	Value
Age of onset back pain, mean (± SD)	29.7 (± 8.7)
Male, *n*	21 (41.2 %)
Marital state	
- Married	15 (45.1 %)
- Unmarried (with partner)	13 (25.5 %)
- Unmarried (without partner)	23 (29.4 %)
Education level	
- Only primary school	3 (5.9 %)
- Secondary education	28 (54.9 %)
- University education	20 (39.2 %)
Duration (months) back pain, mean (± SD)	12.9 (± 5.9)
Work status	
- Employed, *n*	28 (54.9 %)
- Self employed, *n*	7 (13.7 %)
- Domestic work, *n*	8 (15.7 %)
- Studying, *n*	11 (21.6 %)
- Voluntary work, *n*	2 (3.9 %)
- Care taking of family members, *n*	3 (5.9 %)
ASDAS, mean (± SD)	2.3 (± 1.0)
BASDAI, mean (± SD)	4.5 (± 2.6)
BASFI, mean (± SD)	1.7 (± 2.1)

*axSpA* axial spondyloarthritis, *SPACE* Spondyloarthritis Caught Early, *ASDAS* Ankylosing Spondylitis Disease Activity Score, *BASDAI* Bath Ankylosing Spondylitis Disease Activity Index, *BASFI* Bath Ankylosing Spondylitis Functional Index

### Work productivity

When looking at all 51 patients, they worked 36.4 (±13.9) hours and missed 4.2 (± 8.4) hours per week due to axSpA, presenteeism was 28.6 %, absenteeism 8.3 % and WPL 33.7 %. Table 2 shows the hours worked and missed due to axSpA, presenteeism, absenteeism and WPL of patients with paid jobs (employed and self-employed) or performing voluntary work, domestic work, care of family and/or studying. There were 35 patients (68.6 %) in paid work; 6 of them combined paid work with voluntary work (n = 1), domestic work (n = 1), care of the family (n = 2), studying (n = 1) or a combination (n = 1). Patients spent less time (27.6 hours) in daily activities compared to income-earning jobs (35.7 hours). There was no difference between patients involved in paid work and other daily activities in the number of missed hours/week due to the disease. However, while patients in paid work missed <4 hours/week, patients doing domestic work missed >6 hours/week. Patients with paid work had greater productivity loss due to disease (presenteeism = 32.6 % vs 18.6 %, *p* = 0.031). Absenteeism was in general relatively low in this cohort (7.9 % in income-earning patients and 8.3 % in patients performing other activities).

**Table 2:** Work productivity of patients with axSpA according to work status, disease activity (ASDAS/BASDAI), functional ability (BASFI)

	Hours worked per week mean (± SD)	Hours missed per week due to axSpA mean (± SD)	Hours missed per week due to other reasons mean (± SD)	Activity impairment due to axSpA% mean (± SD)	Absenteeism mean% (± SD)	Presenteeism mean% (± SD)	WPL mean% (± SD)
Work status							
Paid work	35.7 (± 12.9)	3.4 (± 6.8)	2.1 (± 3.5)	4.1 (± 2.8)	7.9 (± 14.0)	32.6 (± 31.2)*	36.6 (± 30.0)
- Employed	33.8 (± 9.4)	3.6 (± 7.4)	2.4 (± 3.8)	4.2 (± 2.9)			
- Self employed	43.6 (± 21.4)	2.6 (± 3.3)	1.0 (± 1.7)	3.4 (± 2.4)			
Other daily activities	27.6 (± 12.4)	4.3 (± 9.4)	0.6 (± 1.5)	4.6 (± 3.5)	8.3 (± 13.9)	18.6 (± 28.8)*	25.6 (± 28.5)
- Domestic work	29.1 (± 10.9)	6.4 (± 13.8)	0.8 (± 2.1)	6.1 (± 3.7)			
- Studying	29.0 (± 13.0)	3.7 (± 6.7)	0.4 (± 0.9)	3.6 (± 3.4)			
- Voluntary work	6.0 (0)	-	3.0 (0)	0.5 (± 0.7)			
- Taking care of family members	14.7 (± 9.5)	1.0 (± 1.7)	-	4.3 (± 2.9)			
ASDAS score (disease activity)							
< 1.3 (inactive)	41.4 (± 9.6)	0.8 (± 2.1)	2.8 (± 4.7)	2.6 (± 2.8)	1.7 (± 4.0)*	14.5 (± 21.1)	15.3 (± 22.2)*
1.3–2.1 (moderate)	33.5 (± 18.9)	3.0 (± 6.4)	1.7 (± 3.1)	2.9 (± 2.7)*	5.7 (± 10.0)*	20.9 (± 25.1)	26.3 (± 23.0)*
2.1 − 3.5 (high)	36.9 (± 13.5)	3.3 (± 5.4)	1.2 (± 2.2)	5.2 (± 2.6)*	7.3 (± 10.9)*	32.2 (± 31.2)	36.9 (± 30.1)*
≥ 3.5 (very high)	31.2 (± 10.2)	16.0 (± 16.7)	-	7.0 (± 3.7)	29.2 (± 24.6)*	55.0 (± 44.6)	68.3 (± 28.8)*
BASDAI score (disease activity)							
< 4 (low)	37.6 (± 14.9)	2.3 (± 5.0)	1.9 (± 3.7)	2.5 (± 2.5)***	4.4 (± 8.3)	17.4 (± 24.0)*	20.5 (± 24.4)*
≥ 4 (high)	35.5 (± 13.1)	5.8 (± 10.2)	1.2 (± 2.3)	5.8 (± 2.8)***	11.5 (± 17.0)	37.9 (± 34.1)*	44.5 (± 30.9)*
BASFI score (physical function)							
< 2.5 (low)	35.9 (± 13.5)	2.2 (± 4.4)*	1.8 (± 3.3)	3.6 (± 2.9)***	4.8 (± 8.5)*	21.3 (± 26.2)**	24.8 (± 26.3)**
≥ 2.5 (high)	38.2 (± 15.3)	10.8 (± 13.8)*	0.7 (± 2.0)	6.9 (± 2.6)***	19.6 (± 21.8)*	52.5 (± 36.2)**	62.5 (± 24.7)**

*axSpA* axial spondyloarthritis, *ASDAS* Ankylosing Spondylitis Disease Activity Score, *BASDAI* Bath Ankylosing Spondylitis Disease Activity Index, *BASFI* Bath Ankylosing Spondylitis Functional Index; *p < 0.05; **p < 0.01; ***p < 0.001

### Relationship between work productivity and disease activity or physical functioning

We identified decrease in work productivity alongside increased disease activity (ASDAS). Hours missed at work (*p* = 0.087), absenteeism (*p* = 0.050), presenteeism (*p* = 0.205) and WPL (*p* = 0.012) increased with the progressive increase in disease activity (ASDAS). Patients with very high disease activity (ASDAS ≥3.5) worked on average 10 hours less than patients with inactive disease (ASDAS ≤1.3) (*p* = 0.122). Presenteeism (*p* = 0.049) and WPL (*p* = 0.007) were significantly increased in patients with BASDAI ≥4 compared to patients with BASDAI <4 (Table 2).

Although the number of hours worked were similar (*p* = 0.737), hours missed at work (*p* = 0.021), the degree on disease influence on daily activities (Q6; *p* = 0.020), absenteeism (*p* = 0.014), presenteeism (*p* = 0.010) and WPL (*p* = 0.000) were significantly higher in patients with low BASFI scores (<2.5) compared to patients with high BASFI scores (≥2.5).

Table 3 shows that the associations between work productivity and disease activity and physical functioning were statistically significant on linear regression analyses. Results were adjusted for age, gender, HLA-B27 positivity, duration of CBP. There was no interaction between marital status or educational level and work productivity or BASDAI/ASDAS.

**Table 3:** Relationship between work productivity and disease activity and functional ability in patients with early axSpA

	*R* ^2^	ß-estimated	95 % CI
Absenteeism			
ASDAS	0.30	6.08	2.8–10.8
BASDAI	0.29	2.65	1.1–4.3
BASFI	0.51	4.32	2.9–5.8
Presenteeism			
ASDAS	0.28	16.04	7.1–25.0
BASDAI	0.25	5.80	2.2–9.4
BASFI	0.16	4.62	0.4–8.9
Work productivity loss			
ASDAS	0.36	18.86	10.8–27.0
BASDAI	0.34	7.12	3.9–10.4
BASFI	0.29	7.31	3.6–11.1

Data are adjusted for age, gender, human leucocyte antigen (HLA)-B27 positivity and duration of chronic back pain. *axSp*, axial spondyloarthritis, *ASDAS* Ankylosing Spondylitis disease activity score, *BASDAI* Bath Ankylosing Spondylitis Disease Activity Index, *BASFI* Bath Ankylosing Spondylitis Functional Index, *CI* confidence interval

## Discussion

In this study we found work productivity impairment to be correlated to both disease activity and physical functioning in Italian patients with axSpA with CBP ≥3 months and ≤2 years, with onset  < 45 years of age. In addition, absenteeism was very low compared to that in patients in other countries.

In our study, patients with axSpA with income-earning jobs worked 2.2 hours/week less than the general Italian population with income-earning jobs, who work 36 hours/week (as reported on http://dati.istat.it (June 2015)). We identified 7.9 % absenteeism in patients with paid work, which is similar to the sick leave in the general Italian population (8.4 %). However, the rate of sick leave in our patients with axSpA is much lower than reported in other studies. A Dutch study in patients with early SpA (with <5 years disease duration) reported a rate of 28 % for sick leave in the Amsterdam area [20], a rate almost 7 times higher than in the general population in Amsterdam and 3.5 times higher compared to our data. This difference may be due to the higher average age and the longer disease duration in these Dutch patients with respect to our patients. Moreover, 74 % of Dutch patients had a defined diagnosis of AS, while our patients had nr-axSpA in 92.2 % of cases.

Sick leave rates reported in the literature largely vary between several countries with 47 % in Belgium, 48 % in France and 52 % in the Netherlands, while the sick leave rate was reported as 28 % in Sweden and 16 % in the UK [9, 21, 22]. It has to be pointed out that the great majority of these studies were focused on patients with longstanding AS. In this severely affected patient group, the sick leave rate was expected to be higher due to the proven effect of AS on work productivity [23]. Like absenteeism, presenteeism seems to be lower in our patients compared to other studies. The few available data on presenteeism show rates of 41 % in patients with early SpA and 45 % in patients with AS of 10–18 years disease duration, while we identified a presenteeism rate of 32.6 % in patients with early axSpA [23]. This indicates that Italian patients with early axSpA have reduced work productivity, but the disease does not lead to more sick leave, compared to the general Italian population.

Several studies show correlation between WPL and increased disease activity and decreased physical functioning [9, 24]. In addition, Haglund et al. found BASDAI and BASFI not only to be correlated with presenteeism but also with activity impairment going beyond patients’ paid work [25]. Although those studies focused on patients with longstanding AS, the results of our study were similar, supporting the idea that disease activity more than disease duration can impact on work ability and productivity.

This study was based on a small sample size. Even though the number of patients was relatively small the authors found the results to be of importance, as there are no available data on this matter in Italy. In the future, a longitudinal study could be conducted to more intensively investigate the course of disease activity and its relationship with work productivity. Another limitation of our study was the use of self-report questionnaires to determine disease activity, physical functioning and work productivity.

Self-report questionnaires tend to give a biased view, which can lead to either underestimation or overestimation of the findings. Nevertheless, the validity of these questionnaires was tested and considered good [17]. Therefore validated, standardized questionnaires are often used in these studies and are the preferred tool to assess disease outcome. The strength of our study was the absence of bias due to non-responders, as all 51 patients reported back.

## Conclusions

In conclusion, our analyses showed that absenteeism and presenteeism rates were lower in Italian patients with axSpA with ≤2 years disease duration compared to the rates in other European countries. In addition, in these Italian patients with early axSpA, work productivity correlated with disease activity and physical functioning, as previously was found in in several European studies of patients with longstanding AS.

## Abbreviations

AS: ankylosing spondylitis
ASDAS: Ankylosing Spondylitis Disease Activity Score
axSpA: axial spondyloarthritis
BASDAI: Bath Ankylosing Spondylitis Disease Activity Index
BASFI: Bath Ankylosing Spondylitis Functional Index
CBP: chronic back pain
CRP: C-reactive protein
HLA-B27: human leukocyte antigen-B27
LUMC: Leiden University Medical Centre
MRI: magnetic resonance imaging
nr-axSpA: non-radiographic axial spondyloarthritis
SpA: spondyloarthritis
SPACE: Spondyloarthritis Caught Early
WPAI: work productivity and activity impairment
WPL: work productivity loss
